# Cardiac Strains As a Tool for Optimization of Cardiac Resynchronization Therapy in Non-responders: a Pilot Study

**DOI:** 10.1515/med-2019-0111

**Published:** 2019-12-10

**Authors:** David Šipula, Milan Kozák, Jaroslav Šipula, Miroslav Homza, Jiří Plášek

**Affiliations:** 1 Department of Cardiovascular Diseases, University Hospital in Ostrava, 17. listopadu 1790, 708 00 Ostrava, Czech Republic; 2 Faculty of Medicine, Masaryk University Brno, Kamenice 5, 625 00 Brno, Czech Republic; 3 Clinic of Internal Medicine – Cardiology, University Hospital Brno, 625 00, Czech Republic

**Keywords:** Cardiac Resynchronization Therapy, Heart Failure, Optimization, Speckle Tracking, Cardiac Strains

## Abstract

**Background:**

Approximately 30% of patients do not respond to implantation of Cardiac Resynchronization Therapy – Defibrillators (CRT-D). The aim of this study was to investigate the potential for cardiac strain speckle tracking to optimize the performance of CRT-D in non-responding patients.

**Methods:**

30 patients not responding to Cardiac Resynchronization Therapy-Defibrillators after 3 months were randomly divided into control and intervention groups. Atrioventricular interval was adjusted so that E and A waves did not overlap, the interventricular interval was subsequently optimized to yield maximum improvement of the sum of longitudinal+radial+circumferential strains. The left ventricular ejection fraction (LVEF) and NYHA improvement 3 months after optimization were evaluated and use of other strain combinations assessed.

**Results:**

A significant correlation between the (combined) strain change and LVEF improvement was detected (p<0.01). 75% of patients with non-ischemic etiology of heart failure who did not respond to the original CRT-D reacted favorably with significant LVEF and NYHA improvement. The area strain was the best predictor of LVEF/NYHA improvement in those patients. No significant improvement was recorded in patients with ischemic etiology.

**Conclusions:**

AV and VV optimization based on speckle tracking is a very promising method potentially leading to a significant improvement of the outcome of CRT-D, especially in patients with non-ischemic etiology of heart failure.

## 1 Introduction

If correctly indicated, Cardiac Resynchronization Therapy (CRT) is an efficient non-pharmacological treatment of chronic heart failure. The optimal CRT indication criteria according to the major randomized studies such as PROSPECT, SMART-AV, and ECHO-CRT are: heart failure with NYHA classes II–IV, QRS complex duration over 130ms, chronic left bundle branch block and left ventricular ejection fraction (LVEF) below 35% [1, 2, 3, 4].

However, CRT does not lead to improvement of the functional state in all patients – despite all efforts to identify the patients most benefiting from CRT, approximately 30% of candidates fail to respond [5]. Patients with non-ischemic etiologies of heart failure such as dilated cardiomyopathy (DCMP) were shown to respond more favorably to CRT than patients with heart failure resulting from coronary artery disease (CAD), likely due to formation of scarred tissue in patients with ischemic etiology of heart failure [6, 7]. Cardiologists had long been trying to find methods to identify potential responders or to further optimize CRT to improve the success rate [6, 8, 9, 10].

Speckle tracking is a relatively new tool in echocardiography, allowing acquisition of detailed information on myocardial deformation, expressing it as myocardial strains (defined as the percentage change in length of a segment of myocardium relative to its resting length) [11]. Echocardiography provides 4 types of strains: Longitudinal strain (L), circumferential strain (C), radial strain (R) and area strain (A), which is essentially a combination of the longitudinal and circumferential strains). L, C, and A strains are normally negative while the R strain is usually positive.

In our study, we focused on patients who were not responsive to CRT three months after the original implantation of the cardiac resynchronization therapy-defibrillator (CRT-D) and investigated the potential of speckle tracking for optimization of the CRT performance in those patients. We defined the following research questions: (i) Could re-programming of the interventricular interval on the CRT-D in a way maximizing the sum of cardiac strains (L, C and R strains) affect the CRT outcome in non-responders? (ii) If so, is there any other combination of strains that would predict the outcome better than sum of all strains? (iii) Is there a difference between the response in patients with different etiologies of heart failure, namely DCM and CAD?

## 2 Methods

### 2.1 Study group

This is a prospective cohort study. The research was performed in accordance with the Declaration of Helsinki and approved by the local Ethical Committee. Consecutive patients meeting inclusion and exclusion criteria who presented at our department from 2016 to 2017 and who did not respond to the implantation of CRT-D at the cardiovascular center of the University Hospital in Ostrava three months after the procedure were enrolled in the study. This failure to respond to the original therapy was defined as failure to increase the LVEF by at least 5%, which is generally considered significant [12] and, at the same time, failure to record NYHA improvement. All patients signed an informed consent.

#### 2.1.1 Inclusion criteria

Symptomatic heart failure with NYHA III/IV, pharmacological treatment options exhausted, LVEF below 30%, QRS duration over 130ms and left bundle branch block

#### 2.1.2 Exclusion criteria

Right bundle branch block, significant valvular disease, significant diastolic dysfunction, atrial fibrillation

### 2.2 Study design

Prior to the CRT-D implantation, all patients at our clinic underwent a basic physical examination, ECG, and a 3D echocardiographic examination (Vivid E9, GE Healthcare). 2D Simpson’s biplane method was used for determining LVEF at all stages of the study. NYHA class and usual parameters, such as the dimensions of all heart segments, blood flow through valves, and the kinetics of the right and left ventricles were also recorded. After the initial examination, a biventricular CRT-D was implanted in accordance with guidelines [13]. An electrode was placed into the right atrium, right ventricle, and into the lateral branch of the coronary sinus. After the implantation, the defibrillator was set for the maximal stimulation of the ventricles with the VV interval optimized to reduce the duration of the QRS complex as much as possible. All patients were invited for a follow-up examination in three months. Medication was prescribed in accordance with the valid guidelines.

Patients in whom no response was recorded after 3 months (as mentioned above, response was defined as left ventricular ejection fraction improvement by at least 5% or NYHA improvement by at least 1 class) were deemed non-responders and were enrolled in our study. Those patients were subsequently randomly divided into intervention and control groups using envelope method stratified by etiology (two sets of envelopes were used, one for patients with CAD etiology, another for patients with non-CAD etiology).

In the intervention group, the optimization was performed as follows: First, the atrioventricular interval was optimized according to the blood flow through the mitral valve in order to ensure the best possible filling of the left ventricle. The interval was adjusted in a way ensuring that the E and A waves did not overlap, one following smoothly the other in order to avoid both truncation and fusion. Subsequently, circumferential (C), longitudinal (L), radial (R) and area (A) strains were recorded and their values considered baseline. After that, the interventricular (VV) interval was optimized through adjustments of the VV interval (steps of 10ms) guided by the strain values. After each CRT-D reprogramming, we waited for two minutes prior to recording the strains. The sum of absolute differences between the C, L and R strain baseline values and values after reprogramming was recorded and the VV interval changed by another 10ms, followed by another evaluation of strains. In total, the VV interval was changed in 10ms steps from +10ms up to -40ms in each patient. Finally, the interval providing the highest difference between baseline values of the sum (L+C+R) and values after optimization was used as the CRT-D setting for the particular patient.

Patients in the control group underwent a “sham treatment”, i.e., the same procedure without actually changing any parameters of the CRT-D, to prevent any placebo effect. All recruited patients were advised that they might have undergone either a sham or a real reprogramming.

After three more months, all patients were invited for a follow-up examination during which echocardiography was performed and NYHA class and LVEF were evaluated. The doctor performing the evaluations was blinded to the fact whether or not the patient underwent optimization, thus preventing any bias in evaluation.

## 3 Statistical analysis

Several statistical tests were used to compare the difference in parameters and/or outcomes between control and intervention groups, as well as for comparison of subgroups with different etiologies (CAD vs non-CAD). Continuous variables (age) were assessed by the Kruskal Wallis test (becoming standard Mann-Whitney test where only two samples were compared). For discrete parameters (sex, etiology), the Fisher’s exact test was used. Linear modelling was used to evaluate the regression (explanatory variables – individual and combined changes of strains; dependent variable – change of LVEF). All tests were performed at 0.05 level of significance. Data processing, visualization, and statistical testing was performed in MATLAB and Statistics Toolbox Release 2012b, The Math-Works, Inc., Natick, Massachusetts, United States. Cohen’s d coefficient was calculated to determine effect size.

## 4 Results

Altogether, 30 non-responders were enrolled in the study, 23 of whom were men and 7 women. All patients were Caucasian, median age at initial implantation was 61 years (31-77 years). In the intervention group, the heart failure was of ischemic etiology (CAD) in 7 patients and non-CAD (i.e. dilated cardiomyopathy, DCMP) in 8 patients. In the control group, CAD was the underlying cause in 8 patients and the etiology was non-ischemic in 7 patients. No statistically significant difference was found for any of the descriptive parameters between the control and intervention groups. There were no significant differences in patients’ medication between groups, either.

Table 2 shows the subjective outcome of the optimization. The most notable result is the fact that while NYHA improvement was recorded in six out of eight patients with non-CAD etiology of heart failure after intervention, only one patient with CAD improved after optimization. No improvement of NYHA was recorded in any patient in the control group.

**Table 1:** Comparison of the treatment and control groups

	Group			
	control	intervention	p	Method
Number of patients	15	15	1	Fisher
Sex (Males/Females)	10/5	13/2	0.39	Fisher
Age (min-max)	65 (46-76)	57 (31-77)	0.19	Kruskal-Wallis
Etiology (CAD/DCMP)	8/7	7/8	1	Fisher

**Table 2:** A detailed breakdown of NYHA change before and after intervention in the intervention and control groups as a function of etiology.

		No change or aggravation	Improvement
	All etiologies	8	7
Intervention group	non-CAD	2	6
	CAD	6	1

	All etiologies	15	0
Control group	non-CAD	7	0
	CAD	8	0

**Figure 1:**
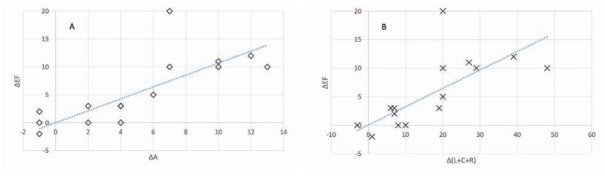
Improvement of the ejection fraction as a function of change in the area strain (A; p<0.001, Pearson coeff. 0.76) and sum of L+C+R strains (B; p<0.001, Pearson coeff. 0.7)

Figure 1 provides a very interesting insight into the relationship between improvements of strains and of LVEF. It is worth noting that we evaluated improvements in all individual strains (and their combinations) and their relationships to LVEF improvement as per Table 3; of the graphs, only the area strain, which provided the best correlation, and the sum of L+C+R strains that was originally intended as the observed parameter, are shown. The graphs show that in all instances where the area strain improved by 6% or more, the LVEF improved by at least 5% and, contrary, where the area strain improved by less than 6%, no significant improvement occurred. The cut-off values for a 5% improvement in ejection fraction and their confidence intervals as derived from the regression analysis are shown in Table 3. In our group, any 5% or greater improvement in LVEF was associated with a NYHA improvement by at least 1, while any improvement below 5% failed to yield any NYHA improvement.

**Table 3:** Regression slopes, values of strain improvement predicting 5% LVEF improvement and p-values for individual strains and their combinations

	Slope		Cut-off for 5% EF improvement			
Strain change	slope estimate	95% CI	Cut-off	95% CI	p-value	Pearson coeff
Δ L	2.42	(1.36 - 3.47)	1.94	(1.88 - 2.01)	< 0.01	0.71
Δ C	1.20	(0.65 - 1.76)	3.89	(3.75 - 4.02)	< 0.01	0.7
**Δ A**	**1.04**	**(0.63 - 1.45)**	**4.57**	**(4.43 - 4.7)**	**< 0.01**	**0.76**
Δ R	0.43	(0.19 - 0.67)	9.38	(8.94 - 9.82)	< 0.01	0.65
Δ (A+R)	0.32	(0.17 - 0.47)	13.88	(13.37 - 14.4)	< 0.01	0.7
Δ (L+C+R)	0.31	(0.17 - 0.46)	15.07	(14.56 - 15.58)	< 0.01	0.7

The change of the area strain provided the most significant results with the narrowest confidence intervals

**Figure 2:**
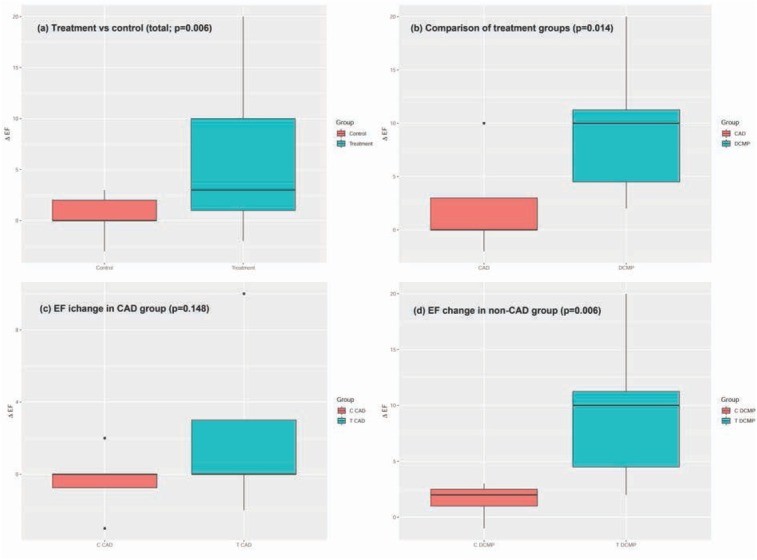
Change of LVEF in the individual categories according to etiology and treatment/control groups; (a) overall treatment (right) vs control (left) group regardless of etiology; (b) treatment groups by etiology (CAD – left, non-CAD – right); (c) control (left) vs treatment (right) group in patients with CAD etiology; (d) control (left) vs treatment (right) group in patients with non-CAD etiology

The differences between groups with different etiologies (CAD vs non-CAD) and treatment vs control groups are shown in Fig. 2. It is obvious that although the LVEF improvement in the treatment group regardless of etiology was significant (Fig. 2a), this effect can be attributed solely to non-CAD patients (Fig. 2b-d). While the difference between the treatment and control groups among CAD patients (Fig. 2c) appears significant, the overall improvement of the EF in the treatment group was, with the exception of a single patient, still below 5%; there was however a notable and statistically significant difference between the non-CAD treatment and control groups (Fig. 2d).

The result of Cohen’s d calculation was 1.17, which means large effect size (>0.8), meaning that despite the small size of our study, the study power was sufficient.

## 5 Discussion

In our prospective pilot study, we optimized the AV interval and subsequently used myocardial strains to optimize the CRT-D performance in a group of patients who did not respond to the original implantation. Despite having a relatively small patient group (30 patients), our method yielded statistically highly significant results with a potential major impact on clinical practice – which of course must be validated in a larger study. Our results however indicate that maximizing the sum of absolute values of strains or the A strain corresponds exceedingly well with the resulting improvement of the left ventricular ejection fraction (and, in effect, with NYHA class improvement).

Reverse remodelling after CRT implantation is a continuous process and in effect, we cannot exclude further changes after three months from implantation [14]. To remove this potential bias, we included a control group of non-responders in whom only sham optimization was performed. As obvious from Fig. 2, however, none of the patients who failed to respond to the CRT-D implantation after 3 months responded even after additional 3 months, unless optimized.

The method originally adopted for this study utilized a simple summation of absolute values of the three principal myocardial strains (i.e., L+C+R strains) and showed a good correlation between the increase of this sum and increase of the ejection fraction. Any improvement of the total of the three cardiac strains by 20 or more resulted in an EF improvement of at least 5% and, at the same time, in NYHA improvement by at least one class within 3 months after the optimization. Contrary, none of the patients with a total L+C+R strain improvement after optimization below 20 recorded a significant improvement of the EF by 5% or more. In this respect, we could actually state that our results indicate a 100% negative predictive value and a 100% positive predictive value of the sum of strains improvement by at least 20 for prediction of the EF improvement by at least 5%. We must however bear in mind that our study group is quite small and hence, we cannot determine such a simple cut-off value based on our patient group. Moreover, there was a gap between the sum of strains improvement in the area of the probable true cut-off point (we recorded no improvement of the sum of strains between 10 and 20 in any one of our patients). Hence, we calculated a confidence interval for the improvement of strains predicting significant EF improvement as 14.56-15.58, see Table 3.

We also analyzed the area strain and all combinations of individual strains and assessed their correlations with EF improvement (Fig. 1, Table 3). Of course, we did not combine the area strain with the L or C strains as the A strain is derived by combining the longitudinal and circumferential strains and such an attempt would create internal dependencies. The results show that although the best predictor of the LVEF improvement was the area strain, all strains and their combinations yielded a statistically significant correlation. Still, the A strain carries the greatest potential for clinical utilization.

Another important result of our study is the significantly better outcome of such optimization in patients with non-CAD etiology over those suffering from CAD. While the optimization was successful (i.e, led to an increase of the ejection fraction by 5% or more) in 75% of patients with non-CAD etiology (6 out of 8), it only led to an improvement >5% EF in a single patient with CAD etiology; it is however notable that the strain improvement in the particular patient was significant and consistent with results of patients with non-CAD etiology who responded well to the optimization. This finding is crucial for selecting patients for such optimization as it appears that patients with non-CAD etiologies benefit from this procedure much more than CAD patients. At the same time, however, it appears that where the strains can indeed be affected by changing the VV interval, the optimization can be effective even in patients with CAD etiology of the heart failure.

Of course, a more detailed breakdown of results in patients with CAD/non-CAD etiologies is needed for better understanding and identification of patients who may benefit from such optimization, this will be however subject of future studies with a much higher number of patients. The same can be said about verification of our results that is necessary for introduction of our results into practice as the results from this pilot study can be only perceived as indicative. We intend to perform such research and would like to encourage anyone interested in joining such a study to contact us.

We appreciate that the number of patients is an obvious limitation of this study. However, as the results are highly statistically significant even in such a small patient group and as collecting a sufficient number of non-responders allowing us to analyze all results and etiologies in detail in a single center setup would take a very long time, we firmly believe that the results of this study are potentially of such significance that they justify early publication as the potential implications are huge. If only EU countries are considered, well over 400 thousand CRT-D implantations were performed in 2013 [15] and it is likely that this number keeps growing. If we discuss patients with non-CAD etiology only who account, according to most multicenter studies, for approx. 30 to 40% of all CRT-D implantations [16], we are discussing over 120 thousand of patients when using the more conservative estimate. The rate of non-responders is generally considered to be approx. 30%, although for example McLeod et al [7] who studied the differences between patients with various etiologies reported the non-response rates to be as high as 47% for patients with CAD etiology and 41% for patients with non-CAD etiology. We would however rather use the results of randomized trials such as CARE-HF and PROSPECT, where the number of CAD responders was 54 and 64%, respectively, while the response among non-CAD patients was higher, namely 79% and 75% [6, 17]. If we remain conservative again and calculate with 21% non-responder rate, we arrive at 25 thousand non-responders with non-CAD etiology annually solely in the aforementioned region and in a major part of those, according to our preliminary results, CRT-D optimization using our method could be successful. Such a potential implication calls in our opinion for publishing our results at the earliest opportunity to allow more extensive research to be performed in this respect and hopefully to bring this method into everyday clinical practice.

Despite the fact that atrial fibrillation is known to be a common condition among the patients with heart failure, we have excluded those patients from our study because it would not allow us to follow the same study protocol (in patients with chronic atrial fibrillation, we would not be able to perform the first optimization step, i.e., the optimization of the AV interval). A study focused on such patients is however also needed to further evaluate the potential contribution of our method in patients with atrial fibrillation.

Another objection to our conclusions could be the combination of optimizing both the AV interval and VV interval based on the strains. It could be argued that the AV interval itself could be a step responsible for the improvement of the outcome. It should be however noted that the same procedure was applied to everyone in the optimized group and it was obvious that regardless of the optimization of the AV interval that was performed in all those patients, some of them responded to the intervention and some did not – and those who did respond were exactly the patients in whom the change in the VV interval led to the increase of the absolute values of the strains. For this reason, we feel confident that utilization of strains is the crucial step in the optimizing procedure, although the AV interval adjustment is deemed to play a role as well.

## 6 Conclusions

In this study, we tested an approach of optimizing the performance of CRT-D in non-responders by changing the AV interval and, even more importantly, by subsequently changing the VV intervals in steps of 10ms until we maximized the absolute value of the L+C+R strains. Our results indicate that the optimizing procedure closely correlated with resulting improvement of the ejection fraction. We also assessed the effect of other strains and their combinations, of which the best result was yielded using the A strain, providing even better results than the L+C+R combination. We also found that such optimization led to a significant LVEF (≥5%) and NYHA improvements in 75% of non-responders with a non-CAD etiology while only in 14% of patients with CAD etiology. It therefore appears that patients with non-CAD etiology are much more likely to benefit from this optimization than CAD patients. Although our results have been only acquired in a small patient group and must be confirmed in a much larger patient group, the high statistical significance even in low numbers justifies our conclusions.

## List of Abbreviations

AV: Atrioventricular CAD - Coronary Artery Disease
CRT-D: Cardiac Resynchronization Therapy – Defibrillator
LVEF: Left Ventricular Ejection Factor
NYHA: New York Heart Association
VV: Interventricular
